# The Structural Binding Mode of the Four Autotaxin Inhibitor Types that Differentially Affect Catalytic and Non-Catalytic Functions

**DOI:** 10.3390/cancers11101577

**Published:** 2019-10-16

**Authors:** Fernando Salgado-Polo, Anastassis Perrakis

**Affiliations:** Oncode Institute and Division of Biochemistry, Netherlands Cancer Institute, Plesmanlaan 121, 1066 CX Amsterdam, The Netherlands; f.salgado@nki.nl

**Keywords:** lysophosphatidic acid, autotaxin, inhibitor, allosteric, orthosteric, lipid chaperone, signalling, GPCR

## Abstract

Autotaxin (ATX) is a secreted lysophospholipase D, catalysing the conversion of lysophosphatidylcholine (LPC) to bioactive lysophosphatidic acid (LPA). LPA acts through two families of G protein-coupled receptors (GPCRs) controlling key cellular responses, and it is implicated in many physiological processes and pathologies. ATX, therefore, has been established as an important drug target in the pharmaceutical industry. Structural and biochemical studies of ATX have shown that it has a bimetallic nucleophilic catalytic site, a substrate-binding (orthosteric) hydrophobic pocket that accommodates the lipid alkyl chain, and an allosteric tunnel that can accommodate various steroids and LPA. In this review, first, we revisit what is known about ATX-mediated catalysis, crucially in light of allosteric regulation. Then, we present the known ATX catalysis-independent functions, including binding to cell surface integrins and proteoglycans. Next, we analyse all crystal structures of ATX bound to inhibitors and present them based on the four inhibitor types that are established based on the binding to the orthosteric and/or the allosteric site. Finally, in light of these data we discuss how mechanistic differences might differentially modulate the activity of the ATX-LPA signalling axis, and clinical applications including cancer.

## 1. Introduction

Lysophosphatidic acid (1- or 2-acyl-*sn*-glycero-3-phosphate or LPA) is a bioactive lipid found in many body fluids and involved in many physiological and pathological processes. Historically, LPA had been identified as a growth factor in serum that could induce motility in fibroblasts and cancer cells through G protein-coupled receptors (GPCRs) [1,2]. Subsequent research identified specific LPA GPCRs (LPA_1–6_), which have distinct expression patterns [3]. Deregulation of the LPA signalling axis has been linked to different diseases, such liver disease [4], fibrosis [5], pruritus [6], multiple sclerosis [7], inflammation, and cancer [8,9].

The LPA receptors are classified into distinct families: the endothelial cell differentiation gene (EDG) (LPA_1–3_) and non-EDG (LPA_4–6_) families. The crystallographic structures of LPA_1_ and LPA_6_ have provided the field with key mechanistic indications with respect to their ligand binding mode [10,11]. Namely, a structural comparison between the LPA_1_ and LPA_6_ substrate-binding sites has indicated contrasting LPA binding modes from the extracellular milieu or the plasma membrane, respectively. The former is consistent with a model by which LPA is carried by a lipid chaperone, such as albumin, to bind to the flexible N-terminal domain of LPA_1_ and deliver LPA specifically [10], whereas the latter would not require lipid-carrying molecules [12,13].

**Figure 1:**
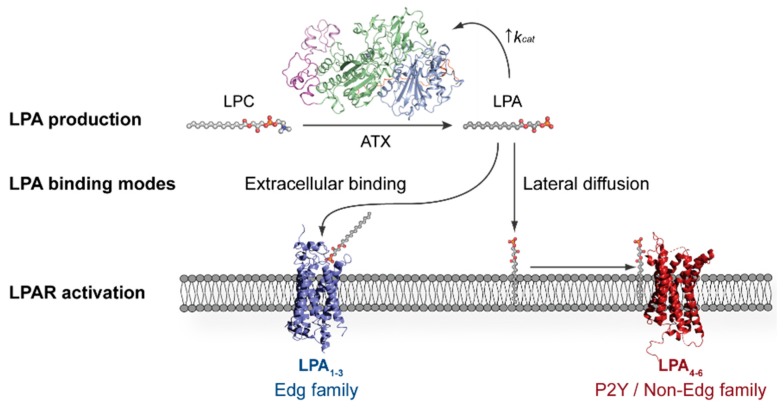
Distinct modes for lysophosphatidic acid (LPA) binding to its cognate G protein-coupled receptors (GPCRs) proposed based on their crystallographic structures. Autotaxin (ATX) is the main producer of LPA, which can then bind to the extracellularly open lipid-binding pocket of LPA_1–3_, potentially assisted by lipid chaperones, or diffuse laterally towards the membrane-open ligand-binding site of LPA_4–6_.

Since LPA can promote a plethora of downstream signalling events, both its production and degradation are tightly regulated, resulting in an estimated half-life of approximately three minutes in circulation [14,15,16]. Such a short-lived existence is due to its fast degradation by three membrane-bound lipid phosphate phosphatases (LPPs), which cleave the LPA phosphate group and release signalling-inactive monoacylglycerol [17,18]. Contrary to this, LPA production originates from the following two sources: phosphatidic acid hydrolysis by PLA_1/2_ and from the enzymatic conversion of lysophosphatidylcholine (1- or 2-acyl-*sn*-glycero-3-phosphocholine or LPC) to LPA by a lysophospholipase D (lysoPLD) that has been established to be Autotaxin (ATX) (Figure 1) [19,20]. ATX-catalysed production constitutes the main physiological source of extracellular LPA, and therefore ATX has been widely studied as a target for drug development [14,21,22].

ATX is the only member of the ectonucleotide pyrophosphatase/phosphodiesterase family (ENPP) that presents lysoPLD activity (EC 3.1.4.39) over lysophospholipids [22]. ATX is first translated as a pre-proenyme that undergoes two proteolytic processing steps, resulting in a mature, glycosylated and secreted form [23]. The determination of the structure of ATX by X-ray crystallography enabled the determination of its domain organization, i.e., the two N-terminal somatomedin B (SMB)-like domains are followed by a central catalytic phosphodiesterase (PDE) domain, which is adjacent to an inactive nuclease-like domain. Substrate hydrolysis requires a bimetallic active site containing two Zn^2+^ ions and a threonine nucleophile, which act in an associative two-step in-line displacement catalytic mechanism [24].

**Figure 2:**
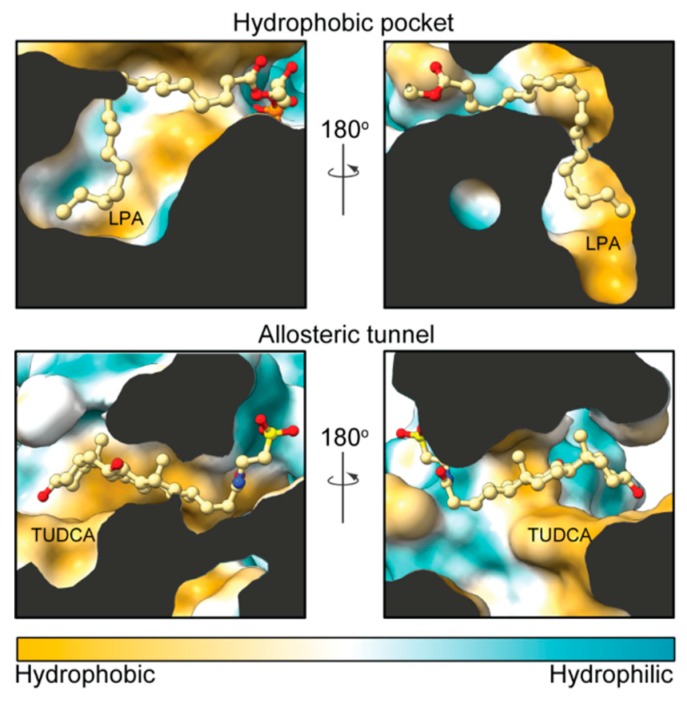
Hydrophobicity comparison between the pocket and the tunnel. Side sections of ATX (Protein Data Bank (PDB): 5dlw) showing the modes of binding of LPA and tauroursodeoxycholic acid (TUDCA) in the orthosteric and allosteric sites, respectively. The partly hydrophobic tunnel presents hydrophilic patches that accommodate the polar moieties of TUDCA. Protein surface was colored from orange to turquois using ChimeraX (version 0.91).

**Figure 3:**
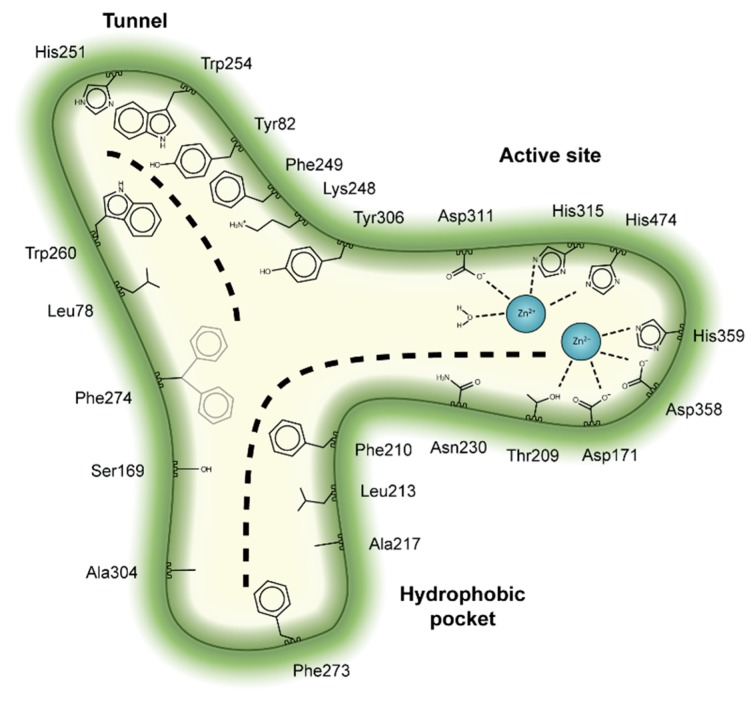
Key residues involved in binding the four classes of ATX inhibitors. Cartoon depiction of the ATX tripartite site and the crucial interacting residues for inhibitor binding. The dashed lines depict the binding site for lysophosphatidylcholine (LPC) or LPA at the orthosteric site, as well as the binding site for steroids at the tunnel.

Structural studies have also established that ATX has a unique tripartite binding site. The catalytic bimetallic site is next to a hydrophilic shallow groove that accommodates the hydrophilic glycerol moiety of lipid substrates. This groove is connected by a T-junction to a hydrophobic pocket where acyl chains can bind, and a tunnel (often called the “hydrophobic channel”) that leads to the other side of the PDE domain [25]. It is noteworthy that the tunnel (or channel) is only partially hydrophobic in nature and has hydrophilic patches, unlike the pocket (Figure 2). The tunnel binds steroid molecules [26], as well as the LPA product [27,28], which results in a modulation of catalytic efficiency. Thus, we refer to the tunnel as the allosteric site, while we refer to the substrate-binding, hydrophilic groove and the hydrophobic pocket, as the orthosteric site (Figure 3).

In this review, we will first review the catalytic mechanism of ATX, especially in light of the allosteric modulation we have recently described, discuss the non-catalytic functions of ATX, and how these are involved in the ATX-LPA signalling axis. Then, we will present the four families of ATX inhibitors from a structural biology perspective, as they are classified depending on their occupancy of the orthosteric and/or the allosteric site. Finally, we will discuss how the different types of inhibitors might interfere with catalytic and non-catalytic functions to differentially affect the ATX-LPA signalling axis in vivo.

## 2. Autotaxin Catalytic and Non-Catalytic Functions

ATX has long been established as the lysoPLD that converts LPC into LPA, but the exact catalytic mechanism remained a subject of study and debate. The first complete characterization of ATX catalysis showed that LPC binding was slow and rate limiting and offered clear evidence for a model where catalysis first results in choline release, which is followed by the slow release of nitrobenzoxadiazole (NBD)-LPA (tens of seconds) [29]. More recently, we have observed an approximately 10 min lag phase in time-course measurements of ATX activity, which could be alleviated by the addition of external LPA [28]. We proceeded to show that LPA binding increases the turnover rate (*k_cat_*) of LPC hydrolysis and promotes its own production. Specifically, our results established that binding of LPA takes place at the low-affinity (~1 µM) allosteric tunnel site. This binding is consistent with earlier results [27] that attributed residual electron density in crystallographic structures of mouse autotaxin to LPA (16:0, 18:0, 18:1, 18:3 and 22:6).

A recent study indicated that active ATX can bind to the surface of secreted exosomes and carry LPA [30]. Such an event could hypothetically lead to LPA-bound ATX at the cell surface, after which ATX would release LPA and activate LPARs. Indeed, the authors indicated that it was by this mechanism that ATX yielded activation of LPA_1_ and LPA_3_ in the employed in vitro experimental setup [30]. However, molecular dynamics simulations and kinetic modelling [28] argue that the presence of LPA in the ATX allosteric tunnel is an independent event, which does not represent an exit pathway of produced LPA by LPC hydrolysis in the adjacent orthosteric site.

The source of the LPC substrate poses another exciting question, that is, its rate of production, lifetime in circulation, and local concentrations in specific organs, which have received rather limited attention compared to the importance of the question. Even though the tunnel does not play a critical role in recruiting LPC substrates from BSA or detergent micelles [27], introduction of different tunnel-occluding loop insertions, based on those present in ENPP1 and ENPP3, resulted in mutants with much impaired cell motility-stimulating activity. Although these observations appear contradictory, the loop insertions could have induced structural changes destabilizing the orthosteric site and resulting in catalytically inactive ATX without just occluding substrate trafficking through the tunnel. Taken together, the evidence is consistent with the role of ATX as an LPA carrier, transporting LPA to distal locations from those where LPC can be taken. LPC is present in the blood at high concentrations (100–200 µM [19]); most of it is bound to serum albumin and cannot be “extracted” by ATX because of their similar affinities (approximately 1 µM) [31], while a small fraction (about 1 μΜ) is free and can be recognized by ATX [20].

Besides its catalysis-dependent functions related to LPA production, ATX also mediates diverse cell signalling events through binding to integrins and heparan sulfate proteoglycans (HSPGs). On the one hand, the more abundant ATXβ isoform binds to integrins, which in turn promotes cell proliferation [32] and directional cell motility [33]. This interaction takes place through the ATX SMB domains, which have high structural similarity with the SMB domain of the cell adhesion factor vitronectin. Specifically, ATXβ can interact with αvβ3 or αIIbβ3 integrins, both of which cause platelet activation upon interaction with ATX [25,34,35]. Accordingly, integrin binding enables uptake and intracellular sequestration of ATX, which redistributes to the front of the migrating cells, however, blockade of integrin binding did not abolish cell migration completely. Interestingly, cell stimulation with only the ATX SMB domains promotes directional cell migration independently of lysoPLD activity [33]. On the other hand, the less abundant ATXα isoform (but not ATXβ) binds to HSPGs through a polybasic insertion [36]. This interaction results in an increase of ATXα catalytic turnover, which may elicit a membrane-anchored burst of LPA production and downstream signalling. Even though this ATXα-specific function lacks further characterization, it has been recently reported that ATXβ can interact in an SMB-independent manner with the HSPG syndecan-4, which affects cell proliferation and cellular metastatic potential [32]. Thus, accumulating evidence suggests several mechanisms for cell receptor binding in the ATX structure, highlighting the relevance of plasma membrane recruitment.

Anchoring on the cell surface is of critical importance in light of the crystallographic structures of LPA_1_ and LPA_6_, which suggested novel mechanisms with respect to their ligand binding modes [10,11]. The LPA_1_ substrate-binding site is located inside a central globular cavity capped by an extracellular N-terminal helical lid. The large (also named “baggy”) ligand-binding pocket remains closed at the membrane side, indicating it is solely accessible from the extracellular space. This enables not only LPA binding, but also potentially phosphorylated endocannabinoids binding and activation of LPA_1_. Such an extracellular lipid-binding site capped by flexible helices is consistent with a model by which LPA is carried by a lipid chaperone, such as albumin, which binds to the flexible N-terminal domain of LPA_1_ and delivers LPA specifically [10]. Conversely, the zebrafish LPA_6_ structure [11] showed a much smaller ligand-binding site, that is not open towards the extracellular milieu, restricting the receptor’s specificity solely to LPA [11]. The LPA_6_ structure is consistent with a binding mode by lateral diffusion of the LPA in the plasma membrane, which would not require lipid-carrying molecules to take place [12,13].

## 3. The Autotaxin Inhibitor Family

Initially, classical ATX inhibitors relied on lipid analogues targeting ATX based on similarities with sphingosine-1-phosphate (S1P) or LPA. Activity-based lead discovery campaigns, using artificial substrates as activity reporters, subsequently made important contributions, many of which are reviewed by Castagna et al. and Nikolaou et al [37,38]. The structural characterization of rat and mouse ATX structures in 2011 [25,27], enabled a remarkable potential for selective inhibitor design by focusing on the three-dimensional architecture of the ATX active site. While attention initially focused on the lipid-binding pocket and the catalytic site, current emphasis is on the so-called tripartite site that we introduced earlier (Figure 3).

**Figure 4:**
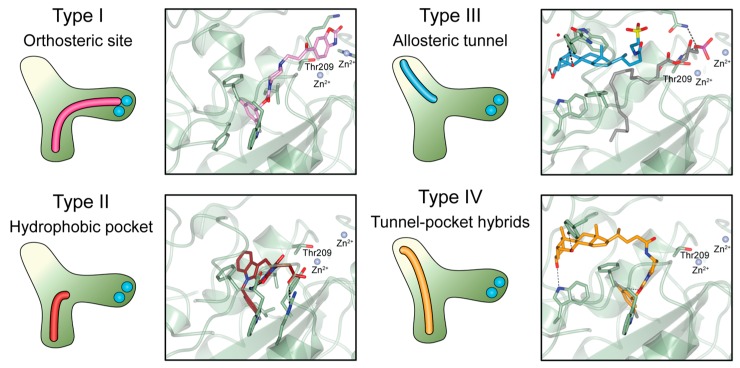
Classification of the four reported ATX inhibitor types based on their binding modes. **Left**: schematic view of the binding modes; **right**: crystallographic structures of ATX bound to compounds from each type; **from top to bottom**: PF-8380 (5l0k), PAT-352 (4zg9), TUDCA (5dlw), and FP-Cpd 17 (5m0m).

The tripartite ATX binding site represents a remarkable potential for selective design of inhibitors. Over time, ATX inhibitors of a distinct chemical nature were designed, including lipid-based inhibitors [39], DNA aptamers [40], as well as small molecules. The last group, the focus of this review, can be in turn classified in four distinct types (I, II, III and IV) depending on their mode of binding to the ATX tripartite site (Figure 4) [41].

In this review we focus on compounds for which there is a crystal structure available. We present, by inhibitor type, the common interactions with ATX (Table 1), known application in in vivo models (Table 2), and discuss their properties in light of their experimentally determined binding pose. It should be noted that due to the unclear naming of some inhibitors as “compound #” in the literature, we will refer to those as “FL-Cpd-#”, where FL stands for the first and last names of the publication’s first author.

### 3.1. Type I Inhibitors

Type I compounds occupy the orthosteric site, mimicking the LPC substrate mode of binding. As such, they include competitive inhibitors with a long and flexible structure that occupies the catalytic site, the shallow groove, and the hydrophobic pocket. Several analogues based on LPA have naturally been put forward as ATX inhibitors such as: cyclic phosphatidic acid or cPA (half maximal inhibitory concentration (IC_50_) = 0.14 µM, bis-*p*NPP) [55], thiophosphate (IC_50_ = 0.6 µM, bis-*p*NPP) [56], α-bromomethylene phosphonates like BrP-LPA (IC_50_ = 0.7–1.6 µM, LPC) [57], the synthetic analogue for S1P FTY720-P (inhibitor constant (K_i_) = 0.2 µM, Bis-*p*NPP) [58], and the most potent lipid-based inhibitor S32826 (IC_50_ = 5.6 nM, LPC) [59]. The latter showed poor in vivo stability, which prevented it from further use in animal models.

The first small molecule type I inhibitors were thiazolidinediones discovered in a screen using the artificial substrate CPF4 [14,25]. Optimization led to the identification of HA-130 (IC_50_ = 28 nM, LPC), a boronic acid-based inhibitor that could attack the nucleophilic oxygen at the catalytic threonine, and it was able to hamper LPA production both in vivo and in vitro [14]. Moreover, a positional isomer of HA-130, HA-155 (IC_50_ = 5.7 nM, LPC) (Table 3), was co-crystallized with ATX, which revealed interactions with Thr209, Asp311, His359 and His474 at the active site, as well as Leu213 and Phe274 in the hydrophobic pocket (Figure 3, Table 1). This series was subjected to structure-activity improvement studies based on the HA-155 crystal structure. These consisted in testing the different linkers added to the thiazolidine-2,4-dione core [60].

Subsequently, numerous type I ATX small molecule inhibitors have appeared in both academic and patent literature. An example of these are the thiazolone derivatives similar to the HA series produced by Kawaguchi and collaborators [63]. A library of 81,600 compounds was screened for inhibiting the hydrolysis of the fluorescence probe TG-mTMP. This led to the identification of KM-Cpd 10 (180 nM, TG-mTMP), 2BoA (580 nM, TG-mTMP), 3Boa (13 nM, TG-mTMP), and 4BoA (22 nM, TG-mTMP), which were co-crystallized with ATX (Table 3). These structures revealed interactions with Arg284, as well as hydrophobic contacts with Trp260 and Phe274 (Figure 3, Table 1). Moreover, these compounds were able to decrease LPA levels both in vitro and in vivo [63].

The most potent type I inhibitor to date is PF-8380 (IC_50_ = 1.7 nM, LPC) (Table 3), reported by Pfizer [61,62]. In general, it has been widely used because of its high potency and its favourable pharmacokinetic properties, which has allowed in vivo evaluation of ATX inhibition. PF-8380 is a benzoxazolone that exhibits the general chemotype of lipophilic tail, core spacer, and acidic head group. This general motif contains a benoxazolone as the acidic head group, a functionalized piperazine as the spacer, and the dichlorocarbamate moiety as the lipophilic portion [61,62]. In the active site, the acidic head group makes essential interactions with one of the Zn^2+^ ions, and the lipophilic tail is accommodated within the hydrophobic pocket. Moreover, it is in close proximity to Thr209 and has hydrophobic interactions with Leu213, Phe273, and Phe274, as well as a hydrogen bond with the Trp275 amino group (Figure 3). The addition of a hydroxyethyl group to PF-8380 resulted in FP-Cpd-3, which had an increased solubility and was co-crystallisation with ATX (Table 3) [65]. ATX inhibition with PF-8380 was shown to attenuate bleomycin-induced pulmonary fibrosis, owing to decreased LPA levels in plasma and bronchioalveolar lavage fluid, together with a decrease in inflammation and collagen deposition (Table 2). However, effectiveness of treatment with this compound varies in the literature [42,66]. Recently, treatment of mice on a high-fat diet with PF-8380 reduced plasma LPA levels, resulting in lower diet-induced cardiac dysfunction and inflammatory response [67].

The compound SBJ-Cpd-1 (IC_50_ = 520 nM, LPC) [7] (Table 3) was obtained from an aminopyrimidine series that was further improved by the addition of the benzoxazolone moiety present in PF-8380. This was crucial for synthesizing the far more active SBJ-Cpd-2 (IC_50_ = 2.5 nM, LPC), which reaches the active site Zn^2+^ ions in a similar manner to that of PF-8380. Additionally, it also contacts Asp171, Asp311, His315 and His474 at the active site, and Leu213, Phe273, Phe274 and Trp275 at the pocket (Figure 3, Table 1). This compound was further tested in rats by a single oral dose (10 mg kg^−1^), where plasma LPA levels decreased 80% upon 12 h treatment (Table 2). Lastly, another relevant compound shown to behave type I inhibitors is the benzotriazole BI-2545 (2.2 nM, LPC) [64] (Table 3), which was able to reduce LPA levels both in vitro and in vivo.

### 3.2. Type II Inhibitors

Type II ATX inhibitors owe their effect solely to binding to the hydrophobic pocket, where they obstruct LPC accommodation. As a result, this competitive mode of binding avoids interaction with the catalytic zinc ions, which may offer selectivity advantages over other inhibitors. The artificial ATX substrate, FS-3, was used by PharmAkea to screen small molecule compounds, from which they identified four indole-based analogues with high inhibitory potency. Among these, three lead type II compounds were reported, namely PAT-078 (IC_50_ = 472 nM; LPC), PAT-494 (IC_50_ = 20 nM; LPC), and PAT-352 (IC_50_ = 26 nM; LPC) [50] (Table 4). The structures of these compounds revealed common hydrophobic interactions between their vinyl-nitrile or hydantoin moieties, and Leu213, Leu216, Phe274, Trp275 and Tyr306 (Figure 3, Table 1).

The artificial ATX substrate FS-3 was also used as readout for a high-throughput screen from a collection of 87,865 compounds. Upon a preliminary selection of 1.2% best hits, the physiological LPC choline release assay was used, from which the imidazo[4,5-b] biyridine-derivative CRT0273750 (IC_50_ = 1 nM, LPC; 14 nM human plasma LPC) [69] was identified (Table 4). The crystal structure of ATX in complex with CRT0273750 indicated that the compound binds at the hydrophobic pocket, as well as with the boundaries of the ATX tunnel, but 5 Å away from the active site. As a consequence, it establishes a hydrogen bond with Lys247, and hydrophobic interactions with Leu213, Phe248, Trp254, Phe273, Phe274 and Trp275 (Table 1). In vitro data showed that this compound could inhibit migration of 4T1 cells. Moreover, the compound was effective in reducing 18:1 LPA levels in vitro in human plasma, as well as in in vivo samples from MDA-MB-231-luc tumour bearing Balb-c nu/nu mice (Table 2) [69]. Lastly, the short phosphonate lipid analogue GWJ-A-23 [68] was used in models for lung fibrosis and inflammation, where it resulted in a 50% decrease of LPA concentrations in bronchioalveolar lavage fluid (Table 2).

### 3.3. Type III Inhibitors

Type III inhibitors specifically occupy the allosteric regulatory tunnel, modulating ATX activity by non-competitive inhibition. We have reported that 7-α-hydroxycholesterol (Table 5) is commonly present in purified mammalian ATX structures, but this does not have an observable inhibitory activity. However, other steroids, namely, bile salts such as tauroursodeoxycholic acid (TUDCA) (IC_50_ = 10.4 µM; LPC) or ursodeoxycholic acid (UDCA) (IC_50_ = 8.8 µM; LPC) exert modest and partial (always leaving residual activity) non-competitive inhibition of lysoPLD activity (Table 5) [26]. The ATX co-crystal, with 18:1 LPA bound at the orthosteric site and TUDCA bound at the tunnel, confirmed the non-competitive mode of inhibition. TUDCA interacts with ATX, forming a hydrogen bond with Trp260 and hydrophobic stacking with Trp254 and Phe274 (Figure 3). The mechanism underlying its inhibition may occur by counteracting the modulatory action of LPA in the tunnel [28].

The identification of type II indole-based ATX inhibitors, as discussed above, also yielded a type III compound, namely PAT-347 (IC_50_ = 0.3 nM; LPC) (Table 5) [50]. The crystal structure of ATX bound to both PAT-347 and 14:0 LPA confirmed its non-competitive binding mode. PAT-347 accommodates at the tunnel by π–π interactions between its indole moiety and Phe274 and His251. Moreover, its benzoic acid makes another π–π interaction with Phe249, and it also contacts Lys248, Trp254, and Trp260. More recently, the pharmacokinetic properties of two novel compounds, PAT-505 (IC_50_ = 2 nM; LPC) and PAT-048 (IC_50_ = 1.1 nM; LPC), have been assessed (Table 5) [70,72,73,74]. The crystal structure of PAT-505 bound to ATX showed a very similar binding mode to that of PAT-347, namely, by interacting with Lys248, Phe249, His251, Trp254, Trp260 and Phe274, but also Ser81 and Val277 (Table 1). Clinical assays with PAT-048, showed better pharmacodynamics results, and this was used for in vivo assays with a bleomycin-induced mouse model for dermal fibrosis. Pharmacological inhibition of ATX activity markedly attenuated skin fibrosis upon treatment with 10 mg kg^−1^ PAT-048, which related to a 75% inhibition of ATX activity after 24 h and >90% at double dose (Table 2). It is worthy to mention that five new patented PharmAkea compounds, specifically, PharmAkea-Cpd A-E (IC_50_ = < 0.5 µM; LPC) have been recently used in metabolic disorder treatment, where they showed a decrease of fasting blood glucose levels in mice fed with a high-fat diet (Table 2) [49].

Recently, another series of indole-derived type III compound has been reported, resulting from a structure-activity evolution of a compound reported by Amira Pharmaceuticals [75], which led to the identification of LM-Cpd 51 [71] (Table 5). The co-crystal structure with rat ATX showed that the compound binds in the tunnel, establishing stacking interactions with Trp254, as well as hydrophobic interactions with Phe249, Trp260 and Phe274 (Table 1).

### 3.4. Type IV Inhibitors

Type IV compounds occupy the binding pocket and the tunnel, but do not contact the catalytic site. Such compounds have been discovered either by design, fusing parts of a type I and a type III inhibitor, such as in FP-Cpd-17 [65], or by serendipity, during a high-throughput screen followed by specific structure-based design, such as in GLPG1690 [51] (Table 6).

We reported the structure-guided design and chemical evolution of bile salts, from weak physiological non-competitive inhibitors into potent competitive type IV compounds [65]. This was achieved by using the PF-8380 dichlorocarbamate moiety as a pocket-binding lipophilic portion. By design, these compounds did not interact with the active site residues, but still hampered LPC or LPA binding. The best lead compounds differed chiefly regarding spacers connecting the steroid and the dichrolocarbamate moieties, i.e., linear amide linker, piperazine, and piperidine for FP-Cpd-5 (IC_50_ = 202 nM; LPC), -11 (IC_50_ = 814 nM; LPC), and -17 (IC_50_ = 20 nM; LPC), respectively (Table 6) [65]. Moreover, a very short spacer yielded no measurable inhibitory activity, which explains why FP-Cpd-11 exhibited a five-fold lower activity than FP-Cpd-17. These compounds were co-crystalized with rat ATX, showing how these types of inhibitors accommodate at both the tunnel and the pocket. Specifically, they all made hydrogen bonds with Tyr81 and Trp260 at the tunnel, and established π–π interactions with Phe273 and Trp274 in the hydrophobic pocket (Table 6, Table 1) [65]. Lastly, these type IV compounds were used in in vitro cell assays, where they showed a decrease of LPA-driven downstream phosphorylation of Akt in BJeH fibroblasts [65].

The Belgo-Dutch company Galapagos NV serendipitously discovered the only other confirmed type IV compound series. They used a high-throughput screen, where they identified an imidazo [1,2-a] pyridine series, from which the very potent compounds GLPG1690 (IC_50_ = 27 nM; LPC) [51] and AJ-Cpd-9 (IC_50_ = 357 nM; LPC) [41] were obtained by structure-activity evolution (Table 6). Moreover, the crystal structures of these compounds confirmed their binding mode to ATX. GLPG1690 makes a hydrogen bond with Trp254, π-π interactions with Phe274, and further hydrophobic interactions with Phe250 and Phe275. Conversely, AJ-Cpd-9 makes a hydrogen bond with Trp260 and hydrophobic interactions with Trp254, Phe250, Phe260 and Phe275 (Table 6, Table 1). After pharmacokinetics and pharmacodynamics analyses [76,77], in vivo experiments showed that administration of GLPG1690 to bleomycin-treated mice resulted in a reduction of lung fibrosis, which was linked to a dose-dependent reduction of plasma LPA 18:2 levels (90%) (Table 2) [41,51]. This compound has shown very promising results in advanced clinical trials against idiopathic lung fibrosis (IPF) and has recently progressed to phase III clinical trials for IPF [78].

## 4. Conclusions

The different types of ATX inhibitors could have different outcomes that go beyond the simple orthosteric inhibition of catalytic activity. While we know that LPA binding to the allosteric site is likely increasing the catalytic rate of ATX, possible roles of the tunnel in delivering LPA to its receptors, perhaps in an interplay between surface integrins and proteoglycans, have not been exploited. The fact that EDG and non-EDG receptors have different binding pocket characteristics could imply that LPA delivery through the tunnel could be receptor-dependent.

Type I inhibitors would effectively stop LPA production from LPC, but would not affect any independent function of the allosteric site. Type IV inhibitors, however, would also abolish any functionality of the allosteric site, expelling bound LPA. We suggest that the clinical success of the type IV compound, GLPG1690, is not independent from its mode of binding and inhibition, interfering with the allosteric tunnel. Albeit some ATX inhibitors have shown promising results in animal cancer models [55,79,80], none have progressed to clinical trials, at least to our knowledge. One should consider the different types of inhibitors in the context of cancer therapy. We suggest that a type IV inhibitor occupying the low-affinity allosteric tunnel could prove much more effective, in the context of a tumour with high local LPA concentration that could displace the inhibitor from the high-affinity orthosteric site. It is our expectation that Autotaxin inhibitors will have a dynamic comeback in the context of cancer therapy.

## Figures and Tables

**Table 1:** Common interactions needed in distinct types of Autotaxin (ATX) inhibitors.

Type		Residues Establishing Ligand Contacts (Rat ATX)		
		Active Site-Hydrophilic Groove	Hydrophobic Pocket	Allosteric Tunnel
I	Common	Thr209, Asp311, His474	-	-
	Frequent	His315, His359	Leu213, Phe273, Phe274 *, Trp275	-
II	Common	-	Leu213, Phe274 *, Trp276	-
	Frequent	-	Phe273, Tyr306	-
III	Common	-	-	Lys248, Phe249, Trp254, Trp260
	Frequent	-	-	Phe274 *
IV	Common	-	Leu213, Phe273, Trp275, Tyr306	Phe249, Trp260
	Frequent	-	Phe274 *, Phe210	His251, Trp254, Phe274 *

* Phe274 sidechain has two predominant conformers at the pocket-tunnel boundary depending on the interacting ligand.

**Table 2:** ATX inhibitors employed in in vivo models. Further details discussed in [21,42,43].

Type	Inhibitor	Disease	LPA Inhibition	References
I	SBJ-Cpd 1	Inflammation Multiple sclerosis	>50%	[7]
I	PF-8380	Glioblastoma Liver fibrosis	>90% >90%	[44] [4]
I	GK442	Pulmonary fibrosis		[45]
I	BMP22	Melanoma metastasis	50%	[46]
II	GWJ-A-23	Pulmonary fibrosis inflammation	50%	[47] [48]
III	PharmAkea -Cpd A-E	Metabolic disorder	25–35%	[49]
III	PAT-505, PAT-048	Liver fibrosis Skin fibrosis	40–80%	[50]
IV	GLPG1690	Pulmonary fibrosis Clinical trials in IPF patients	84–95% 84–95%	[51] [52]
**?**	ONO-8430506	Breast cancer Thyroid cancer	>60% >70%	[53] [54]

**Table 3:** Type I ATX inhibitors based on their kinetic and crystallographic analysis. * denotes that TG-mTMP was used as the substrate for reporting the IC_50_.

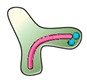	Type I Inhibitor	PDB ID	Activity (IC_50_)	Reference
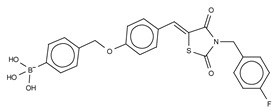 **HA-155**		2xrg	5.7 nM	[25,60]
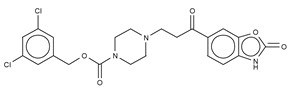 **PF8380**		510k	1.7 nM	[61,62]
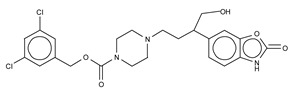 **FP-Cpd 3**		5m0e	-	[62]
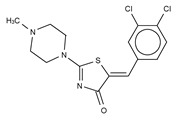 **MK-Cpd 10**		3wav	180 nM *	[63]
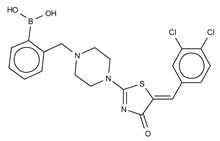 **2BoA**		3waw	580 nM *	[63]
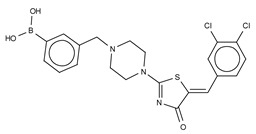 **3BoA**		3wax	13 nM *	[63]
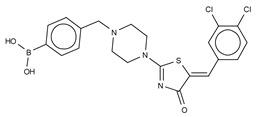 **4BoA**		3way	22 nM *	[63]
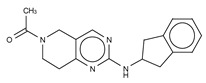 **SBJ-Cpd 1**		5l0b	520 nM	[61]
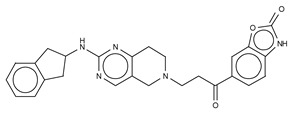 **SBJ-Cpd 2**		5l0e	2.5 nM	[61]
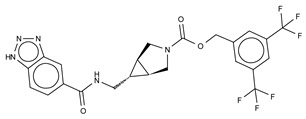 **BI-2545**		5ohi	2.2 nM	[64]

* TG-mTMP used as subtrate.

**Table 4:** Type II ATX inhibitors based on their kinetic and crystallographic analysis.

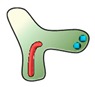	Type II Inhibitor	PDB ID	Activity (IC_50_)	Reference
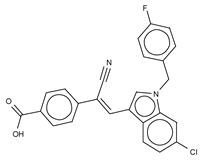 **PAT-078**		4zg6	472 nM	[50]
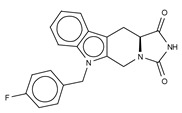 **PAT-494**		4zga	20 nM	[50]
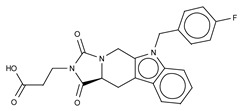 **PAT-352**		4zg9	26 nM	[50]
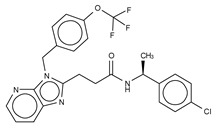 **CRT0273750**		5lia	1 nM	[68]

**Table 5:** Type III ATX inhibitors based on their kinetic and crystallographic analysis.

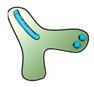	Type III Inhibitor	PDB ID	Activity (IC_50_)	Reference
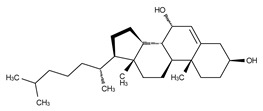 **7-α-hydroxycholesterol**		5dlt	undetermined	[26]
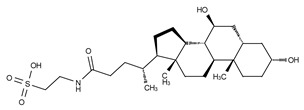 **TUDCA**		5dlw	10.4 µM	[26]
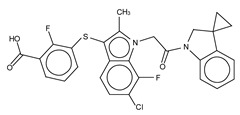 **PAT-347**		4zg7	0.3 nM	[50]
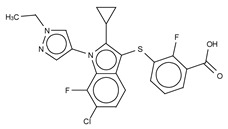 **PAT-505**		5kxa	2 nM	[70]
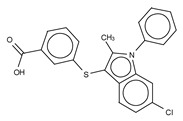 **LM-Cpd 51**		5lqq	81 nM	[71]

**Table 6:** Type IV ATX inhibitors based on their kinetic and crystallographic analysis.

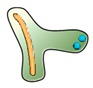	Type IV Inhibitor	PDB ID	Activity (IC_50_)	Reference
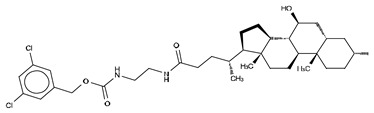 **FP-Cpd 5**		5m0s	202 nM	[65]
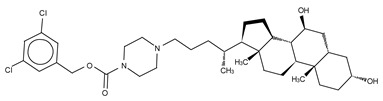 **FP-Cpd 11**		5m0d	814 nM	[65]
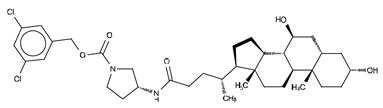 **FP-Cpd 17**		5m0m	20 nM	[65]
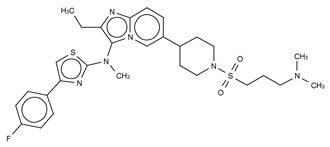 **AJ-Cpd 9**		5m7m	357 nM	[41]
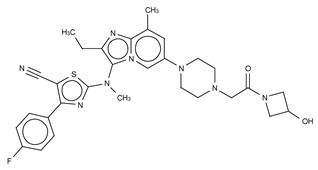 **GLPG1690**		5mhp	27 nM	[51]
